# Role of Artificial Intelligence in TeleStroke: An Overview

**DOI:** 10.3389/fneur.2020.559322

**Published:** 2020-10-07

**Authors:** Faryal Ali, Umair Hamid, Osama Zaidat, Danish Bhatti, Junaid Siddiq Kalia

**Affiliations:** ^1^Department of Medicine, Dow University of Health Sciences, Karachi, Pakistan; ^2^Department of Neurology, University of Illinois, College of Medicine, Peoria, IL, United States; ^3^Departments of Endovascular Neurosurgery and Stroke, St. Vincent Mercy Medical Center, Toledo, OH, United States; ^4^Department of Neurological Sciences, University of Nebraska Medical Center, Omaha, NE, United States; ^5^AINeuroCare, Dallas, TX, United States; ^6^Clinical Strategy, VeeMed Inc., Roseville, CA, United States

**Keywords:** artificial intelligence, machine learning, telestroke, teleneurology, telehealth, telemedicine

## Abstract

Teleneurology has provided access to neurological expertise and state-of-the-art stroke care where previously they have been inaccessible. The use of Artificial Intelligence with machine learning to assist telestroke care can be revolutionary. This includes more rapid and more reliable diagnosis through imaging analysis as well as prediction of hospital course and 3-month prognosis. Intelligent Electronic Medical Records can search free text and provide decision assistance by analyzing patient charts. Speech recognition has advanced enough to be reliable and highly convenient. Smart contextually aware communication and alert programs can enhance efficiency of patient flow and improve outcomes. Automated data collection and analysis can make quality improvement and research projects quicker and much less burdensome. Despite current challenges, these synergistic technologies hold immense promise in enhancing the clinician experience, helping to reduce physician burnout while improving patient health outcomes at a lower cost. This brief overview discusses the multifaceted potential of AI use in telestroke.

## Introduction

Stroke is the second most common cause of mortality and disability worldwide (1). Risk of death and poor prognosis after a stroke increase with delays in diagnosis and treatment. At the same time, there has been a growing shortage of neurologists, particularly in underserved rural areas (2). This shortfall in supply is expected to increase from 11% in 2012 to an estimated 19% by 2025, with the most pronounced deficit being among vascular neurologists (2, 3). While there exists many contributing factors, recent surveys have found that as many as 60% of neurologists report at least one symptom of burnout or depression, one of the highest rates when compared to other specialties (4, 5).

With a limited diagnostic and therapeutic window and a growing shortage of specialized clinicians, there is a need to come up with innovative ideas in stroke management for the purpose of improving patient outcomes. Teleneurology is one example of a technological innovation that has been successful in helping to improve stroke outcomes in the last two decades (6). However, challenges remain in terms of workload and efficiency, including time needed for assessment, lack of trained physicians, burden of documentation, and risks of errors.

Artificial intelligence (AI) is another game-changer that has the potential to reduce some of these barriers to care. This brief overview summarizes the various ways in which artificial intelligence has become incorporated into the field of vascular neurology, with a focus on the potential benefits of its incorporation into telestroke.

## Defining Telehealth, Telemedicine, Teleneurology, and Telestroke

Telehealth is the delivery of health care at a distance using information and communication technology. Telemedicine enables clinicians to provide direct patient care at a distance by leveraging telecommunication information technology (7). Teleneurology is a branch of telemedicine that allows neurologists to care for patients who may not otherwise have access or who may have difficulty making in-person appointments due to mobility issues (8). Telestroke, one of the first and currently most extensive application of this technology, is the use of telemedicine to provide care in acute stroke and neurocritical care settings (9).

### Impact of Telestroke

With the advancement of technology and its increasing accessibility, the use of teleneurology continues to be on the rise today, particularly in rural settings (10). Studies on the impact and feasibility of outpatient teleneurology has so far been promising, with reports of high patient satisfaction, along with the potential for cost savings (10). By improving the ease of access to neurological care, patients living in medically underserved areas are more likely to receive timely and consistent care. This helps to address the relative physician shortage that often leads to delayed diagnoses and treatment, while improving the continuity of care by reducing unnecessary patient transfers and redundant testing (11). By making neurological care more readily available, teleneurology has the potential to not only decrease costs but also to improve patient outcomes.

### Time Is Brain

When dealing with acute neurological problems, time is of the essence, with latency in care being associated with significantly poorer patient health outcomes (12). Here, telestroke provides a compelling alternative to traditional, in-person care. Studies have shown that the accuracy of telestroke in diagnosing acute stroke is equivalent to that of bedside evaluations (13). The importance of this technology's role in allowing immediate assessments is even recognized in the 2018 guidelines for acute ischemic stroke care (14). Using synchronous audiovisual clinical evaluations and diagnostic neuroimaging reviews, telestroke has the ability to efficiently make diagnostic decisions and determinations for IV alteplase candidacy while also providing guidance for thrombolysis administration. Additionally, it can help triage patients who may be eligible for a mechanical thrombectomy that may require inter-facility transfer (15).

## Artificial Intelligence In Stroke Management

### AI in Acute Stroke Imaging

Artificial intelligence algorithms using machine learning can be used to automate the reading and classification of radiologic imaging where efficiency and quality of care depend on the rapid interpretation of clinical data, such as in the Emergency Department. Rapid calculations of stroke location and severity can directly impact management decisions and assist in designating prognosis. Deep learning algorithms have the ability to identify time-sensitive abnormalities such as intracranial hemorrhages, its subtypes (i.e., intraparenchymal, intraventricular, subdural, and subarachnoid), in addition to calvarial fractures, midline shift, and mass effect (16). Automated triaging of CT and MRI scans by AI algorithms has the potential to not only optimize physician workflow but also improve patient outcomes through faster detection of acute abnormalities (17).

Studies on computer-aided Alberta Stroke Program Early CT Score (ASPECTS) have shown high sensitivities in the early detection of ischemic changes after being trained on only a limited number of brain scans (18), with diagnostic accuracy surpassing that of a human reader, especially earlier on (between 1 and 4 h mark) (19). Beyond the 4-h mark, the algorithmic approach performs equally well when compared to a human reader.

In the case of large vessel occlusion ischemic strokes, it has been shown that patients who are beyond 6 h of initial symptom presentation benefit from endovascular perfusion therapy (20). These patients must meet perfusion imaging selection parameters. In response, many stroke centers are incorporating the use of automated perfusion processing software to interpret perfusion raw data (21).

### Continuous Automated Outcome Prediction

In addition to enhancing the diagnostic process, AI has been shown to hold immense potential in automating prognosis and calculating precise outcomes. It can predict 3-month treatment outcomes by analyzing physiological parameters during the first 48 h after a stroke (22). This technology has also been known to predict the outcomes of acute ischemic stroke after intra-arterial therapy (23). Additionally, it can be used to predict the severity of cognitive impairments after a stroke as well as the course of recovery over time (24).

## Artificial Intelligence In Clinical Decision Support

### Intelligent EMR

Intelligent electronic medical records (EMR) is one of the newest innovations in medical record keeping. AI is being used to read through an entire EMR using a technique called natural language processing (NLP). NLP aids the conversion of conversational text into a structured representation which enables the automatic identification and extraction of information (25). NLP has been used in the past by neurologists to analyze unstructured data from EHR, including progress notes and neuroradiology reports. Garg et al. used NLP to classify patients into ischemic stroke subtypes with good results (26). Intelligent EMR can also be an essential tool for telestroke in that it can inform vital lab results, review past history for details that would affect management (e.g., atrial fibrillation or recent surgery), and calculate scoring for various stroke scales using designated parameters.

Box 1 **A Glimpse into the Future—AI-Enhanced TeleStroke.**

The emergency medical technician responds to a stroke call for a 55-year-old man at three in the morning. They perform a prehospital stroke scale that yields a score of more than 2, which alerts the ER physician. At the same time, the stroke attending physician is awakened by an alert notifying him of a possible large vessel occlusion. Before the patient even reaches the hospital, the intelligent EMR sifts through the patient's records, creating a pertinent summary for the providers. The EMR simultaneously alerts the telestroke attending, who is currently 50 miles from the hospital, of the patient's history of atrial fibrillation and the medications he is taking for it. Meanwhile, the patient is negative for brain bleed on Computed Tomography (CT) imaging and undergoes a CT Angiography (CTA). The software suggests a Tissue Plasminogen Activator (tPA) bolus, and the attending accepts the suggestion. It further suggests a CT Perfusion (CTP) along with a CTA but the attending only decides on the CTA. The software reads the CTA as it is being performed and detects a large vessel occlusion. It instantly informs the neuro-interventionist while reporting back to the telestroke attending. While performing these tasks, the AI application calculates and gives outcome predictions based on the stroke scale score, imaging reports, and EMR data it has received. The neuro-interventionist performs a thrombectomy after which the telestroke attending is notified automatically. The entire communication is carried out via HIPAA-compliant AI software.

### Documentation, Quality, and Workflow Enhancement Using AI

Automated note creation using speech recognition software has already been made possible, in which machine learning allows the physician to convert speech into text, saving time in the process (27). Another innovation facilitating telestroke physicians is automated timestamping of EMR events. Additionally, creating an automated summary for providers using intelligent EMR helps to wrap up the care process in a timely fashion.

Along with enhancing communication, another role of AI is in optimizing workflow and decision-making. Communication remains an administrative burden to physicians, contributing to burnout, delayed care, and missed care opportunities. Most physician schedules are now online, and all patient-related information can be imported into algorithms to create an organized and streamlined workflow process.

Similarly, AI algorithms can be integrated into various steps in the management of acute strokes, thus streamlining this critical process. Pre-hospital notifications can be sent to the emergency department, alerting staff to the possibility of an incoming stroke and/or large vessel occlusion from data gathered from the patient's signs and symptoms. Notification of bleeds or automated ASPECTS rating can also be sent to appropriate parties (i.e., neurosurgery and neurocritical care vs. stroke and neurointerventional team).

Another potential role of AI is in data collection. There is a significant burden of collecting quality data for acute stroke patients. AI can work in the background to automatically collect data for quality assessment and through metric analyses, subsequently guide quality improvement measures to help meet guidelines.

### Contextually Aware Communication

Managing hospital communications and information can be challenging. At the same time, acute neurology is intense and time-critical. It requires a high level of coordination between physicians, nurses, and technicians who may be located in multiple locations. Two-way pagers and secure messaging have been used to improve communications, but such methods have their own limitations due to changing staff and situations involving concurrent neurological emergencies. A context-aware system will consider staff scheduling, timing, roles, as well as patient location and status. AI algorithms can be designed to have a sense of contextual awareness with which teleservice can help bring clarity and reduce the likelihood of errors.

### Challenges and Limitations

For all the benefits that telestroke has to offer, there are still some challenges and limitations that need to be overcome. Interoperability poses the most significant challenge to integration. We need to break the data silos and have a universal and secure information-exchange highway in order to provide cost-effective and efficient care to patients while at the same time preserving the clinician experience and avoiding moral injury.

The need for broadband access and connectivity to fast, reliable internet is another limitation of effective AI integration in a telestroke program. AI must be operated at the edge and not merely in the cloud. Additionally, a wireless internet infrastructure must be optimized in both WiFi (indoors) and 5G (mobile).

## Conclusion

Despite existing challenges, coupling the technologies of AI and telestroke has the potential to create a seamless experience for both patients and clinicians. By reducing delays in care during neurological emergencies through augmenting the physician's armamentarium, this technology has the potential to streamline workflow, decrease burnout, and enhance the overall provider experience. AI in telestroke will pay for itself as the increased productivity and greater efficiency translate into better patient outcomes. Most importantly, AI is an evolving technology that will continue to strengthen over time, giving this platform endless potential for improving patient care. We look forward to the day when the neurologist can use AI-integrated telestroke to take better care of patients.

## Author Contributions

FA: initial draft and research. UH: second draft and research. OZ, DB, and JK: outline, concept, and editing. All authors: contributed to the article and approved the submitted version.

## Conflict of Interest

JK was employed by the companies AINeuroCare and VeeMed Inc. The remaining authors declare that the research was conducted in the absence of any commercial or financial relationships that could be construed as a potential conflict of interest.
